# Epigenetic and O-glycosylation regulation of p66Shc mitigates mitochondrial oxidative stress in aortic dissection

**DOI:** 10.7150/thno.124508

**Published:** 2026-02-04

**Authors:** Wenjun Zhang, Wanjun Liu, Xiaodan Zhong, Lei Dai, Xiaolei Liu, Shiliang Li, Hongcheng Jiang, Xingwei He, Wei Dong, Lijuan Lu, Li Zhu, Thati Madhusudhan, Hongjie Wang, Hesong Zeng

**Affiliations:** 1 Department of Cardiology, Tongji Hospital, Tongji Medical College, Huazhong University of Science and Technology, Wuhan 430030, Hubei, China.; 2 Hubei Provincial Engineering Research Center of Vascular Interventional Therapy, Wuhan 430030, Hubei, China.; 3 Division of Cardiothoracic and Vascular Surgery, Tongji Hospital, Tongji Medical College, Huazhong University of Science and Technology, Wuhan 430030, Hubei, China.; 4 Division of Hepato-Pancreato-Biliary Surgery, Tongji Hospital, Tongji Medical College, Huazhong University of Science and Technology, Wuhan, Hubei 430030, P. R. China.; 5 Hubei Key Laboratory of Hepato-Pancreato-Biliary Diseases, Wuhan, Hubei 430030, P. R. China.; 6 Clinical Medicine Research Centre for Hepatic Surgery of Hubei Province, Hubei 430030, P. R. China.; 7 Key Laboratory of Organ Transplantation, Ministry of Education; NHC Key Laboratory of Organ Transplantation; Key Laboratory of Organ Transplantation, Chinese Academy of Medical Sciences, Wuhan, China.; 8 Cyrus Tang Medical Institute, Soochow University, Rm 509, Bldg 703, 199 Ren'ai Road, Suzhou 215123, China.; 9 Center for Thrombosis and Hemostasis, Johannes Gutenberg University Medical Center, Mainz, Germany.; 10 Department of Cardiology, Tongji Xianning Hospital, Xianning 437011, Hubei, China.

**Keywords:** aortic dissection, p66Shc, YB1, O-glycosylation, mitochondrial oxidative stress

## Abstract

**Background:** Aortic dissection (AD) is a life-threatening vascular emergency with limited effective pharmacological treatments. Recent studies have identified Src homology 2 domain-containing transforming protein C1 (p66Shc) as a crucial mediator of oxidative stress, apoptosis, and inflammation in aortic cells, thereby contributing to cellular dysfunction and vascular remodeling implicated in AD development and progression. Despite its established role in promoting vascular dysfunction and remodeling, the protective potential of targeting p66Shc in AD remains unclear.

**Methods:** We quantified activated protein C (aPC) levels in clinical plasma samples from control subjects and AD patients using enzyme-linked immunosorbent assay (ELISA). To evaluate changes in p66Shc expression, we analyzed aortic tissues by Western blotting (WB), immunohistochemistry (IHC), and immunofluorescence (IF) staining. An *in vivo* AD model was established in thrombomodulin (TM)-mutant ApoE^-/-^ mice, which display impaired TM-dependent PC activation, and exogenous PC was administered to evaluate its therapeutic effect. In parallel, mechanistic studies were performed in human endothelial cells using WB, co-immunoprecipitation (Co-IP), dual-label IF staining, chromatin immunoprecipitation (ChIP), luciferase reporter assays, and mitochondrial functional analyses.

**Results:** In this study, we demonstrate that aPC, a coagulation protease with known cytoprotective properties, downregulates *p66Shc* expression through epigenetic modifications. Additionally, aPC can modulate the expression of a cold shock protein Y-box-binding protein 1 (YB1), which acts as a transcription factor, leading to elevated O-linked N-acetylglucosamine transferase (OGT) levels. This upregulation enhances the O-glycosylation of p66Shc on its 29th tyrosine residue, preventing its mitochondrial translocation, preserving mitochondrial membrane potential, and reducing reactive oxygen species (ROS) production. Consequently, these molecular mechanisms inhibit the onset and progression of AD.

**Conclusions:** aPC epigenetically represses p66Shc transcription and promotes its O-glycosylation at Thr29 *via* the YB-1/OGT axis, thereby inhibiting mitochondrial ROS production and preventing vascular injury.

## 1. Introduction

Aortic dissection (AD) is a catastrophic cardiovascular condition associated with high in-hospital mortality. Its incidence has been rising in parallel with population aging and uncontrolled hypertension 1, 2. Despite advancements in surgical and interventional techniques, no pharmacological strategies have been clinically validated to prevent the onset or progression of AD. Consequently, early diagnosis followed by emergent surgical or endovascular repair remains the mainstay of treatment 1. However, the molecular mechanisms and risk factors underlying AD initiation and propagation remain incompletely understood.

Recent studies have identified elevated levels of reactive oxygen species (ROS), matrix metalloproteinases (MMPs), and pro-inflammatory mediators as critical contributors to extracellular matrix (ECM) degradation and vascular wall instability in AD pathogenesis 3. Among the emerging regulators of oxidative stress and inflammation is activated protein C (aPC), a serine protease derived from its precursor protein C, traditionally known for its anticoagulant function 4.

Beyond hemostasis, aPC exerts pleiotropic protective effects on the vasculature 4. It was originally recognized in sepsis studies that aPC can reduce inflammation and prevent endothelial injury, which led to its clinical use in severe sepsis 5. Mechanistically, these cytoprotective effects are mediated by aPC binding to the endothelial protein C receptor (EPCR) and activation of protease-activated receptor-1 (PAR1) on endothelial cells 6. In contrast to the pro-inflammatory signals of thrombin-PAR1, the EPCR/PAR1 ligation by aPC initiates biased signaling that stabilizes endothelial barriers, inhibits apoptosis, and dampens inflammatory cytokine expression 7. Moreover, in various disease models such as diabetic nephropathy, aPC has demonstrated significant antioxidant effects, independent of its anticoagulant activity, prompting clinical interest in engineered aPC variants with preserved cytoprotective properties but reduced bleeding risk 8.

Given the prominent role of mitochondrial ROS in vascular injury, we hypothesized that aPC may exert protective effects against AD by modulating mitochondrial oxidative stress 9. A central regulator of mitochondrial ROS is the adaptor protein Shc, encoded by the Shc1 gene, which produces three isoforms: p46Shc, p52Shc, and p66Shc. Among these, p66Shc is uniquely involved in Redox signaling due to its additional N-terminal CH2 domain, which is essential for mitochondrial ROS generation and apoptosis 10.

p66Shc has emerged as a critical mediator in several cardiovascular pathologies, including ischemia-reperfusion injury, endothelial dysfunction, and vascular remodeling. Notably, the regulation of p66Shc itself is complex, involving both transcriptional/epigenetic controls and post-translational modifications 11. The p66Shc promoter is subject to epigenetic regulation, which can restrict its expression to certain cell types or conditions 12, 13. For instance, suppression of p66Shc gene expression by SIRT1-dependent deacetylation or by specific microRNAs has been shown to protect endothelial function in hyperglycemia 14, 15. Once expressed, p66Shc protein activity is modulated by multiple post-translational modifications. Phosphorylation at Ser36 by PKC-β or JNK is required for its mitochondrial translocation, and this process is enhanced by upstream modifications such as acetylation at Lys81 11. Indeed, acetylation of p66Shc at Lys81 increases Ser36 phosphorylation and thus boosts its mitochondrial ROS-generating capacity 11. Conversely, redox modifications like cysteine sulfhydration can dampen p66Shc activation, illustrating the finely tuned control of this “stress adaptor” protein 16. Importantly, emerging data suggest that O-linked glycosylation (O-GlcNAcylation) may also regulate p66Shc and related stress signaling pathways. O-GlcNAcylation is a nutrient-sensitive modification on serine/threonine residues that often crosstalks with phosphorylation 17. Under chronic stress (e.g. diabetes), aberrant O-GlcNAcylation contributes to mitochondrial dysfunction and impaired cardiac performance 17, 18. However, dynamic O-GlcNAc modifications can also serve as protective signals in acute stress by modulating key proteins' activity and localization 17, 18. It is plausible that O-GlcNAcylation of p66Shc (or its upstream regulators) might inhibit its pro-oxidant action, acting as a brake on ROS production.

Based on these insights, we hypothesize that aPC may protect against AD by epigenetically and/or post-translationally regulating p66Shc expression and function. In the current study, we investigated whether aPC modulates mitochondrial ROS production and vascular injury in a p66Shc-dependent manner, thereby providing a mechanistic link between aPC signaling and AD pathogenesis.

## 2. Materials and Methods

### 2.1. Human aortic dissection (AD) and control samples

A total of 27 patients diagnosed with AD and 29 healthy controls were recruited. Clinical parameters, including sex, age, history of hypertension, diabetes, smoking status, drinking alcohol status and ACEI/ARB Drugs were obtained from electronic medical records (Table S1). For AD patients, peripheral blood was collected within 12 hours of hospital admission, prior to surgical intervention. Plasma was separated via immediate centrifugation and stored at -80 °C. Aortic tissue samples were collected during surgery (aortic root/ascending aorta replacement or descending aortic stenting) and processed for histopathology. AD diagnosis was confirmed by experienced cardiothoracic surgeons and verified through histological evaluation. Control aortic tissues were obtained from the Biospecimen Bank at Tongji Hospital. This study was conducted in accordance with the ethical principles of the Declaration of Helsinki and approved by the Ethics Committee of Tongji Hospital, Tongji Medical College, Huazhong University of Science and Technology (approval number: TJ-IRB202410058). All specimens were anonymized to protect participant confidentiality, and data were stored securely in accordance with institutional guidelines. Procedures involving human tissues adhered to relevant national and international regulations governing biomedical research.

### 2.2. Animal studies

Animal experiments were approved by the Institutional Animal Research Committee of Tongji Medical College and conducted in accordance with Directive 2010/63/EU. TM^p/p^ (E404P mutation of the thrombomodulin) mice were purchased from Cyagen Biosciences, and ApoE^⁻/⁻^ mice from Beijing Vital Laboratory Animal Technology. TM^p/p^ × ApoE^⁻/⁻^ mice were generated *via* crossbreeding. Male mice (8 weeks old) were assigned to four groups: saline, protein C (PC), angiotensin II (Ang II), and Ang II + PC. All mice received 0.1% β-aminopropionitrile (BAPN) in drinking water for 3 weeks, followed by 2-week infusion of either saline or Ang II (2,500 ng/kg/min) *via* osmotic minipumps. PC (1 mg/kg) was administered intraperitoneally daily for 2 weeks. Mice were maintained on a 12-hour light/dark cycle at 23 °C with free access to food and water 19.

### 2.3. Enzyme-linked immunosorbent assay (ELISA)

Human and mouse plasma aPC and insulin levels were measured using ELISA kits (Cusabio, Wuhan, China). Samples and standards were added to antibody-precoated plates, incubated at 37 °C for 30 minutes, washed, and incubated with enzyme-labeled detection antibodies. After a chromogenic reaction and termination, absorbance at 450 nm was recorded. Concentrations were calculated from standard curves. All samples were tested in duplicate 6.

### 2.4. Cell culture and treatment

HUVECs, Eahy.926, and 293T cell lines (ATCC) were cultured in RPMI-1640 or DMEM supplemented with 10% FBS at 37 °C in 5% CO₂. Serum starvation was applied for 6 h before treatments. aPC was administered prior to Ang II to determine optimal treatment conditions. Lepirudin (1 μg/mL) was added to eliminate thrombin-related effects. For degradation assays, CHX (10 μg/mL) and actinomycin D (5 μM) were used to block protein synthesis and RNA transcription, respectively 6.

### 2.5. Western blotting

Proteins were extracted using RIPA buffer and quantified using the BCA method. After SDS-PAGE and transfer to PVDF membranes, blots were blocked with 5% BSA and probed overnight at 4 °C with primary antibodies against p66Shc, ICAM-1, VCAM-1, AcH3, YB1, OGT, Flag, MMP2, MMP9, and GAPDH. Secondary HRP-conjugated antibodies were used for signal detection *via* ECL. Densitometry was performed using ImageJ.

### 2.6. Quantitative real-time PCR (qRT-PCR)

Total RNA was extracted using TRIzol and reverse-transcribed to cDNA. qPCR was performed using the Applied Biosystems 7900HT system. GAPDH served as the internal control. Primer sequences are listed in Table S2.

### 2.7. Dual-luciferase reporter assay

293T cells were co-transfected with pGL3-OGT promoter constructs, pRL-TK (internal control), and YB-1 overexpression plasmids. Luciferase activity was assessed using the Dual-Luciferase Reporter Assay System (Promega) per manufacturer's protocol. Reporter constructs spanning various OGT promoter regions and mutated sequences were used to define YB-1 binding 6.

### 2.8. Reporter plasmid construction

OGT promoter (~2 kb) and its truncated or mutated variants were synthesized and cloned into pGL3-basic vectors using XhoI and HindIII sites. Site-directed mutagenesis was performed with the Quick Mutation Kit (Beyotime). Primer sequences are detailed in Tables S4 and S5 6.

### 2.9. Immunoprecipitation (IP) and immunoblotting

Protein lysates were precleared with A/G agarose and incubated with antibodies against p66Shc or Flag at 4 °C, followed by pulldown with protein A/G agarose overnight. Immunoprecipitates were analyzed by SDS-PAGE and Western blotting.

### 2.10. Histology, immunohistochemistry, and immunofluorescence (IF)

Tissue samples were paraffin-embedded, sectioned (4 μm), and stained with H&E or EVG. Immunohistochemistry used primary antibodies (p66Shc, α-SMA, CD68, MMP2, MMP9, gp91) and DAB visualization. IF was performed with antibodies against p66Shc and CD31, followed by DAPI nuclear staining and fluorescence microscopy.

### 2.11. Cellular immunofluorescence

HUVECs or Eahy.926 cells were stained with MitoTracker Red, fixed, permeabilized, and incubated with primary antibodies overnight. After secondary antibody incubation, nuclei were counterstained with DAPI. Images were acquired with an Olympus fluorescence microscope. Colocalization was quantified in five random fields.

### 2.12. DHE staining for ROS detection

Cells were pretreated with PC or aPC (2 nM), followed by Ang II (1.0 μM) for 24 h. DHE probe was applied, and fluorescence intensity was analyzed by ImageJ under a fluorescence microscope 20.

### 2.13. Mitochondrial ROS and membrane potential assays

MitoTracker Red CMXRos (200 nM) was used to evaluate mitochondrial ROS. JC-1 dye was used to assess mitochondrial membrane potential (MMP). Red-to-green fluorescence shifts indicated MMP depolarization 20.

### 2.14. Chromatin immunoprecipitation assay

Chromatin immunoprecipitation was performed as previously described. HUVECs were cultured in 100 mm dishes with 6 mL complete culture medium and transfected with the viruses of high-expression YB1-Flag protein. Paraformaldehyde was added directly into dishes at the final concentration of 1% and incubated at 37 °C for 10 min to get cross-linked. By adding 0.8 mL glycine solution (10X), cross-link was quickly stopped. Then, cells were washed with cold PBS with 1mM PMSF and collected into centrifuge tubes. We used 0.2 mL SDS Lysis Buffer with 1mM PMSF to suspend around one million HUVECs. Ultrasonic cell disruptor was employed to cut genomic DNA into 250-750 bp fragment. After the preparation of ChIP samples, ChIP dilution buffer with 1 mM PMSF was added to a final volume of 2 mL. For precipitation, 20 μL samples were used as input control. Almost 2 mL samples were precleared with 40 μL protein A/G agarose at 4 °C for 1 h to avoid unspecific binding. Cleared samples were then incubated with 1 μg antibody to Flag/Histone 3 or irrelevant IgG antibody at 4 °C overnight. 60 μL protein A/G agarose beads were used to precipitate the complex. Complex was washed with low salt immune complex wash buffer, high salt immune complex wash buffer, LiCl immune complex wash buffer and TE buffer carefully and separated at elution buffer. We used 20 μL 5M NaCl to separate the protein and genomic DNA cross-linking and purified it with an assay kit (Beyotime). ChIP samples were stored at -80 °C or used for real time qPCR immediately. The presence of immunoprecipitated DNA sequence around-1,351/-1,343 bp was detected by quantitative PCR 6. Primer sequences used in ChIP was listed in Table S3.

### 2.15. Statistical analysis

Statistical analyses were performed using GraphPad Prism (version 9.4.1). Data are presented as mean ± SEM. Differences between two groups were assessed using unpaired Student's *t*-tests. Comparisons among three or more groups were evaluated using one-way or two-way ANOVA, followed by appropriate post-hoc tests where applicable. A *P* value < 0.05 was considered statistically significant.

## 3. Results

### 3.1. Exogenous administration of protein C alleviates aortic dissection

**Figure 1:**
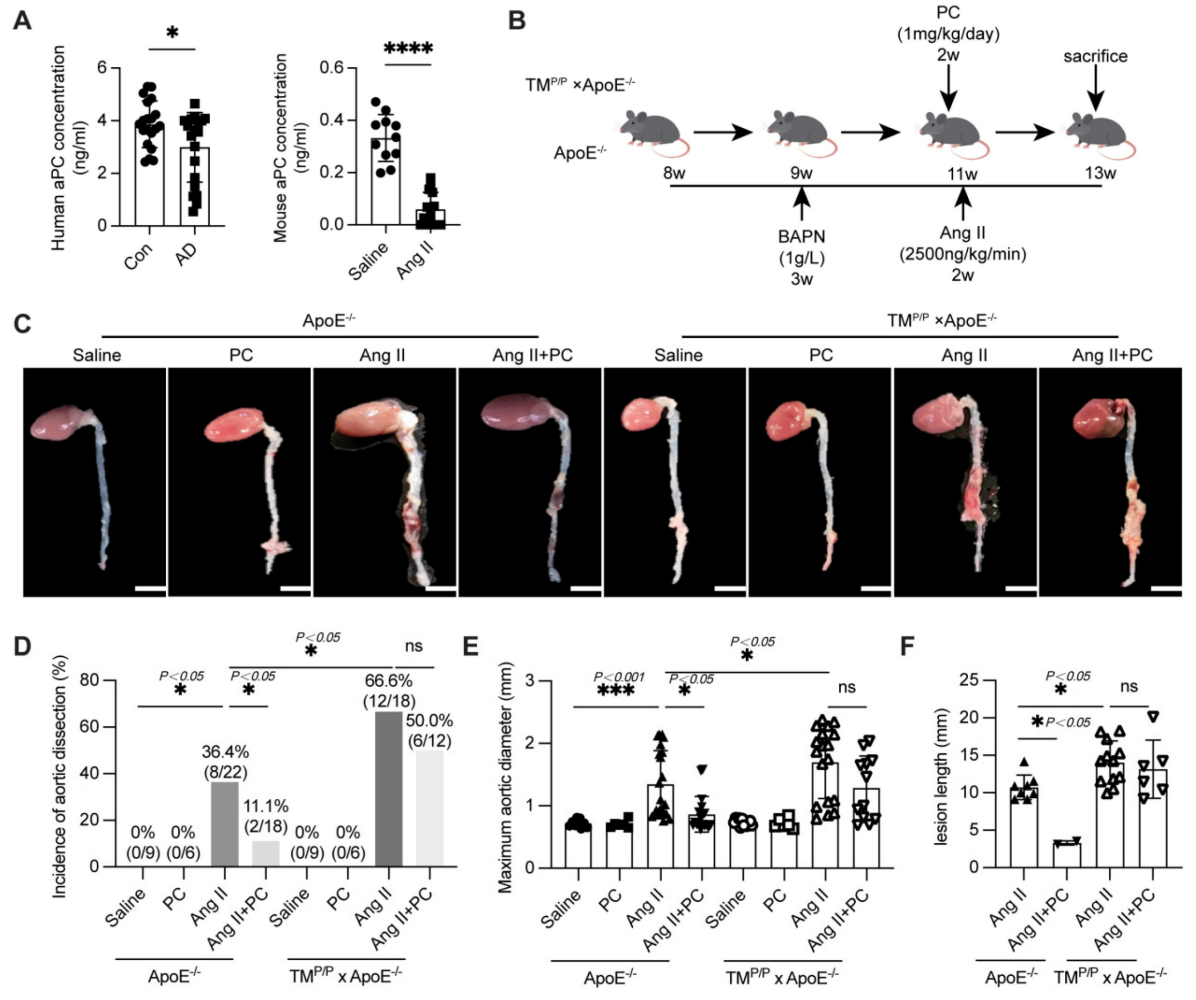
** Exogenous administration of PC through* in vivo* activation into aPC has a protective effect on aortic dissection**. (A) The content of aPC in the human plasma of the control group and aortic dissection group in human and mouse was detected by ELISA, and the results showed that the content of aPC in the plasma of the aortic dissection group was significantly lower than that of the control group. The changes in aPC concentration in mice are consistent with those in humans (n >5/group; *P < 0.05 vs. control or saline group). (B) Schematic illustration of the AD experimental mouse model. 8- week-old ApoE^-/-^ and TM^P/P^×ApoE^-/-^male mice were given β-aminopropionitrile (BAPN) at a concentration of 0.1% for 3 weeks at first and infused with saline or Ang II (2500 ng/kg/min) via subcutaneous osmotic minipumps for 2 weeks. One group received PC 1 mg/kg/day intraperitoneally at the same as the Ang II osmotic minipump was implanted. (C) Representative images of aortae isolated from ApoE^-/-^ and TM^P/P^×ApoE^-/-^ mice in different groups. (D) The occurrence of aortic dissection in each group showed that administering exogenous PC to the Ang II group of wild-type mice effectively reduced the incidence of aortic dissection; The incidence of aortic dissection in the Ang II group of TM^P/P^ type mice is higher than that in the wild-type Ang II group, and administering exogenous PC does not reduce the incidence of aortic dissection. (E) The maximum diameter of each group's aorta showed that administering exogenous PC to the Ang II group of wild-type mice can effectively reduce the maximum diameter of the aorta; The maximum diameter of the aorta in the Ang II group of TM^P/P^ type mice is larger than that in t the Ang II group of wild-type mice, and administering exogenous PC cannot reduce the maximum diameter of the aorta. (F) The range of involvement in mice with aortic dissection showed that administering exogenous PC to the Ang II group of wild-type mice can effectively reduce the range of aortic dissection; The range of aortic dissection injury in the Ang II group of TM^P/P^ type mice is larger than that in the Ang II group of wild-type mice, and administering exogenous PC in the Ang II group of TM^P/P^ type mice cannot effectively reduce the range of aortic dissection. (All data represent the means ± SEM, n ≥ 6; *P <0.05 **P < 0.01, ***P < 0.001; ns indicates no statistically significant; One-way ANOVA).

Activated protein C (aPC) is widely recognised for its anti-inflammatory, anti-apoptotic, and cytoprotective properties in ischaemic injury 6. To explore its relevance to aortic dissection (AD), we first measured circulating aPC in patients and in Ang II-treated mice. ELISA revealed a significant reduction in plasma aPC levels in both human AD subjects and Ang II-infused mice compared with their respective controls (Figure 1A). Several mechanisms may contribute to the reduced aPC levels observed in both human and murine AD tissues. First, the intense oxidative stress and inflammatory response characteristic of AD markedly downregulate thrombomodulin (TM) expression on the endothelial surface, thereby impairing the ability of thrombin-TM complexes to aPC 21, 22. Reduced TM availability directly limits endogenous aPC generation. Second, during AD progression, extensive thrombus formation within the false lumen consumes circulating aPC through binding and proteolytic activity within the coagulation cascade. This consumption-driven depletion has been described in other thrombotic states and is supported by clinical and experimental observations 23, 24.

We next evaluated whether restoring aPC limits AD formation. ApoE^-/-^ mice and thrombomodulin-mutant ApoE^-/-^ littermates (TM^P/P^ × ApoE^-/-^, which display impaired TM-dependent PC activation) were subjected to a well-established BAPN + Ang II protocol 19. Beginning in week 11 of Ang II infusion, mice received daily intraperitoneal PC (1 mg/kg) or vehicle for two weeks (Figure 1B). In wild-type ApoE^-/-^ mice, Ang II alone produced a 36.4 % (8/22) AD incidence, an enlarged maximal aortic diameter (1.35 ± 0.11 mm), and an extensive dissection length (10.73 ± 0.58 mm). PC supplementation dramatically lowered these indices to 11.1 % (2/18), 0.86 ± 0.07 mm, and 3.30 ± 0.21 mm, respectively (Figure 1C-F). By contrast, TM^P/P^ × ApoE^-/-^ mice exhibited a markedly higher baseline susceptibility: Ang II induced AD in 66.6 % (12/18) of animals, with maximal diameter 1.70 ± 0.14 mm and dissection length 14.05 ± 0.83 mm. Exogenous PC failed to confer full protection in this setting (Dissection incidence 50.0 %, 6/12; maximal diameter 1.28 ± 0.15 mm; dissection length 13.16 ± 1.59 mm; Figure 1C-F).

Collectively, these data show that conversion of PC to aPC is essential for vascular protection: exogenous PC limits Ang II-driven AD in mice capable of normal PC activation, but offers only partial benefit when TM-mediated activation is impaired.

### 3.2. Exogenous protein C preserves medial architecture and dampens inflammatory remodeling in Ang II-induced AD

**Figure 2:**
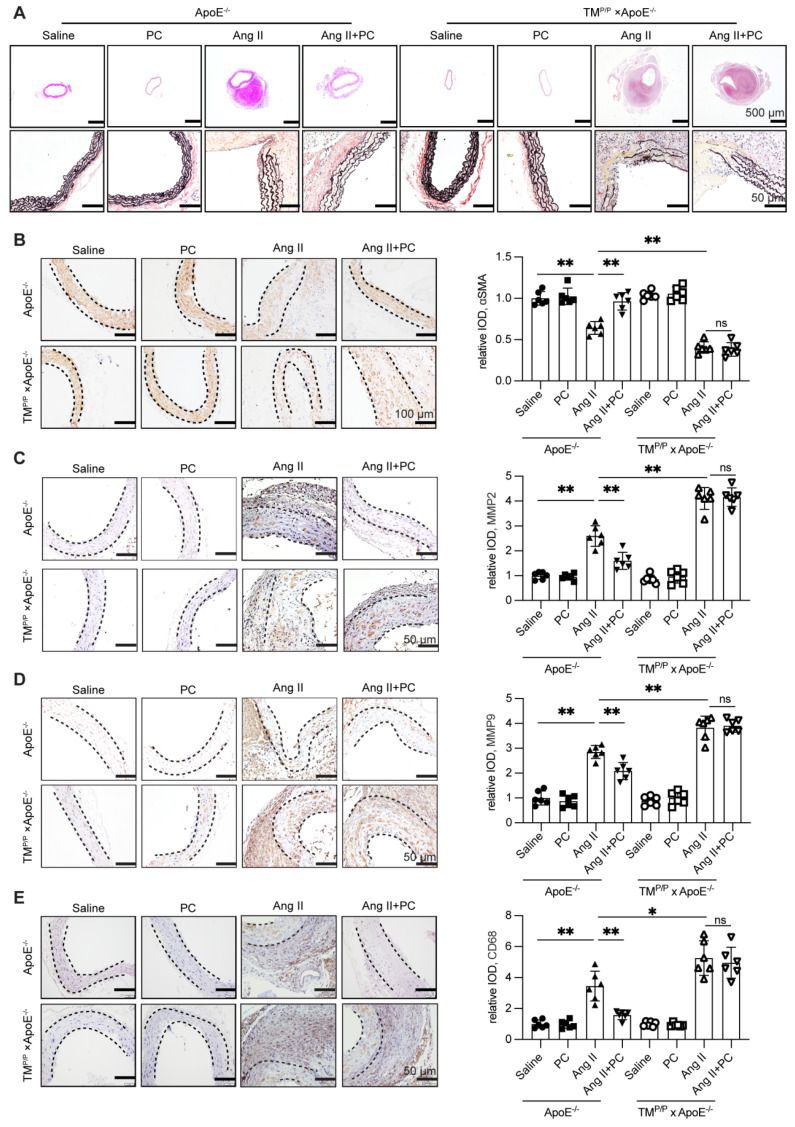
** Exogenous administration of PC can effectively reduce the damage to the middle layer of aortic dissection vessels and inflammatory infiltration.** (A) H&E, α-SMA, MMP2, MMP9, CD68 immunohistochemical and elastin van Gieson staining of aortae with different interventions are shown. (B-E) Quantitative analysis of α-SMA, MMP2, MMP9 and CD68. (All data represent the means ± SEM; n = 6 mice in each group; *P < 0.05 vs. *P < 0.05 vs. saline group, Ang II+PC group in wild-type mice, Ang II+PC group in TM^P/P^ type mice; ns indicates no statistically significant; One-way ANOVA).

Histological analyses corroborated the functional protection afforded by aPC. In wild-type ApoE^⁻/⁻^ mice, Ang II infusion caused hallmark dissection changes—marked luminal dilatation, extensive fragmentation of elastic lamellae (Verhoeff-Van Gieson), loss of α-SMA-positive smooth-muscle cells, adventitial thinning, and dense CD68⁺ macrophage infiltration—accompanied by strong up-regulation of MMP-2 and MMP-9 (Figure 2A-E;Supplementary Figure S7). Daily protein C supplementation almost completely reversed these alterations: elastic fibers remained largely intact, the medial layer retained α-SMA staining, adventitial thickness was preserved, and both CD68⁺ cell density and MMP-2/-9 signals were markedly reduced (Figure 2A-E; Supplementary Figure S7).

In contrast, TMᴾ/ᴾ × ApoE^⁻/⁻^ mice displayed even greater elastic disruption, medial loss, macrophage accumulation, and MMP-2/-9 expression after Ang II infusion. Exogenous PC failed to ameliorate any of these indices (Figure 2A-E; Supplementary Figure S7), underscoring that the histological protection depends on efficient *in vivo* conversion of PC to aPC.

Together, these data show that functional aPC signaling is required to maintain medial integrity, restrain matrix-degrading enzymes, and curb inflammatory infiltration during Ang II-induced AD.

### 3.3. p66Shc expression is upregulated in AD and correlates with increased ROS and MMP-2/-9 expression

**Figure 3:**
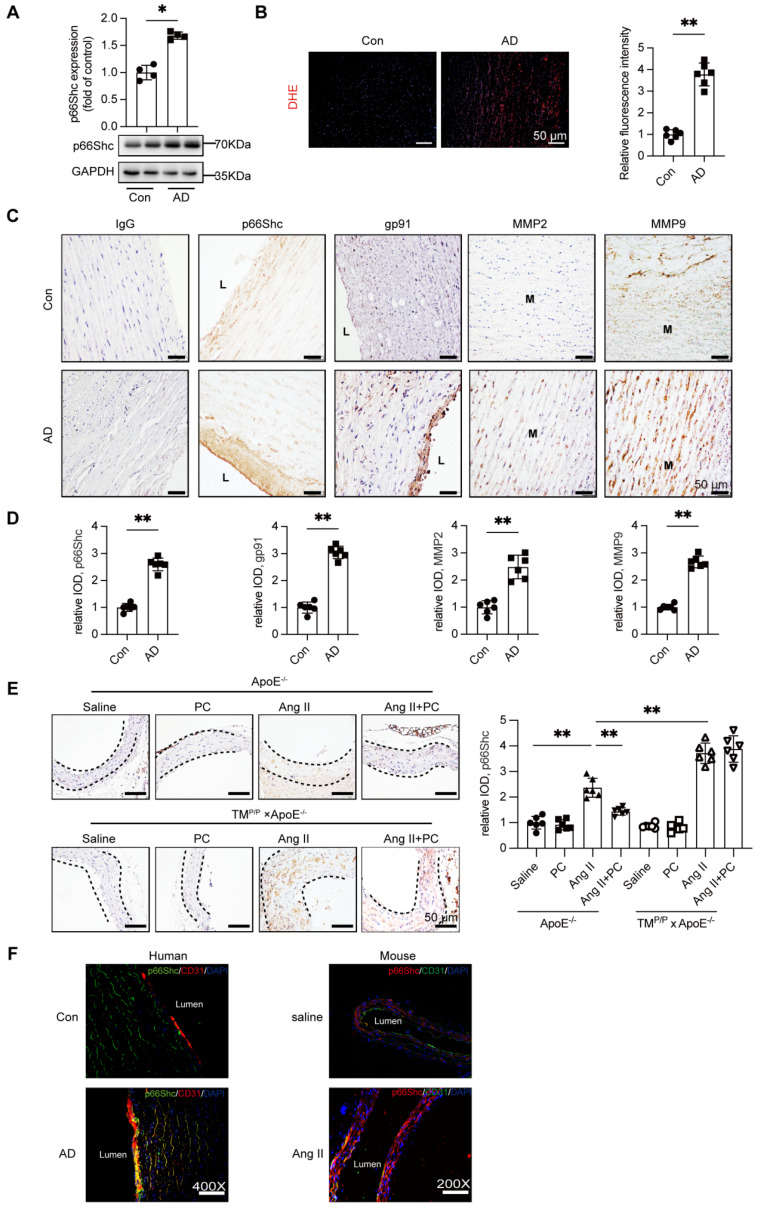
** Increased expression of adapter protein p66Shc in aortic tissue of patients with aortic dissection accompanied by elevated ROS levels and expression of MMP2/9.** (A) Western blot was used to detect the expression of p66Shc protein in the aortic tissue of the control group and aortic dissection group, and the expression of p66Shc protein was significantly increased in the aorta of patients with aortic dissection. (B) DHE fluorescence staining was used to detect the levels of ROS in the control group and aortic dissection group (red represents ROS, blue represents nucleus), and the results showed an increase in ROS levels in the aorta of patients with aortic dissection. (C) Immunohistochemical staining was used to detect the expression of p66Shc protein, oxidative stress indicator gp91, and MMP2/9 in the aortic tissue of the control group and aortic dissection group. The results showed that the expression of p66Shc protein, oxidative stress indicator gp91, and MMP2/9 was significantly increased in the aorta of aortic dissection patients compared to the control group. (D) Quantitative analysis of p66Shc, gp91 and MMP2/9. (E) p66Shc immunohistochemical staining of aortae with different interventions are shown. (F) Double-labeled immunofluorescence assay was used to detect expression of p66Shc in human and mouse aortae. Green represents the p66Shc protein in human or mouse aortae, red represents the endothelial cell marker CD31 protein in human or mouse aortae, blue represents the nucleus, and yellow represents the localization of p66Shc onto endothelial cells. (All data represent the means ± SEM; n ≥ 4 mice in each group; *P < 0.05 vs. control, saline group, Ang II+PC group in wild-type mice, Ang II+PC group in TM^P/P^ type mice; ns indicates no statistically significant; One-way ANOVA).

APC has been shown to down-regulate the redox adaptor p66Shc in diabetic nephropathy 12; given the central role of ROS in AD, we examined p66Shc expression in human AD tissue. Western blotting of ascending-aorta samples demonstrated a marked increase in p66Shc protein in AD patients relative to healthy controls (Figure 3A). Consistently, DHE staining revealed significantly greater ROS accumulation in the dissected aortae (Figure 3B).

Immunohistochemistry confirmed robust p66Shc immunoreactivity in AD specimens, with prominent endothelial-cell localization that paralleled intense staining for the NADPH-oxidase subunit gp91^phox^ and for the matrix-remodelling enzymes MMP-2 and MMP-9 (Figure 3C-D).

The mouse model yielded similar findings. In wild-type ApoE^⁻/⁻^ mice, Ang II infusion elevated aortic p66Shc expression, whereas daily PC supplementation substantially blunted this increase. In TM^P/P^ × ApoE^⁻/⁻^ mice Ang II induced even higher p66Shc levels, and exogenous PC failed to reduce them (Figure 3E). Dual immunofluorescence further showed that p66Shc signal co-localized exclusively with the endothelial marker CD31 in both human and murine AD tissue (Figure 3F; Supplementary Figure S9).

Together, these results identify vascular endothelium-specific induction of p66Shc as a hallmark of AD, tightly associated with enhanced ROS production and up-regulation of MMP-2/-9. Moreover, effective down-regulation of p66Shc requires functional activation of protein C to aPC* in vivo*.

### 3.4 aPC suppresses Ang II-driven p66Shc signalling, matrix remodelling, and oxidative stress through epigenetic modifications

**Figure 4:**
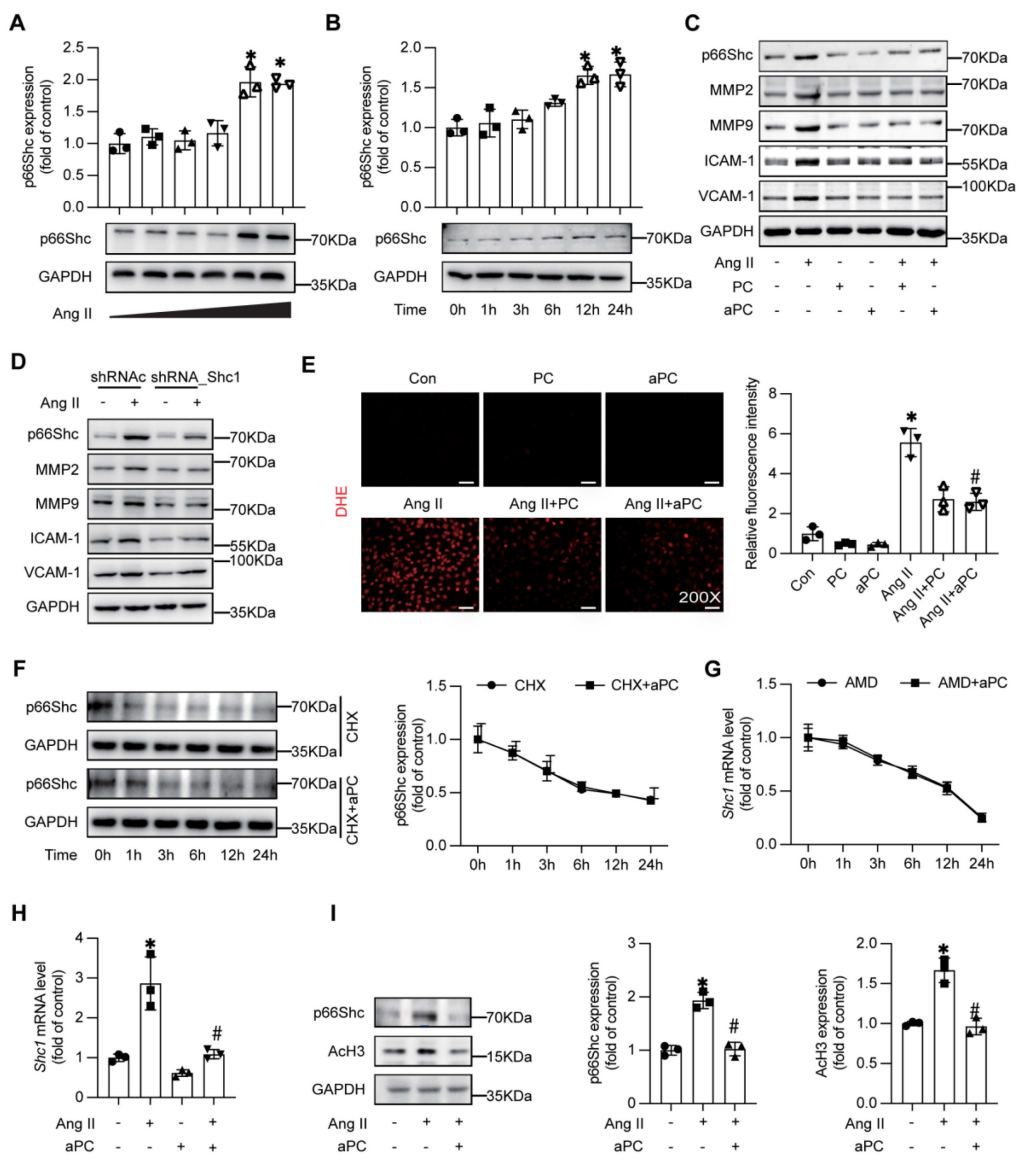
** aPC can inhibit Ang II induced p66shc, MMP2/9, inflammatory markers, and oxidative stress levels, and reduce p66Shc protein expression by regulating histone acetylation.** (A-B) Western blotting shows the expression of p66Shc was induced by Ang II in a concentration and time dependent manner. (C) Intervention with Ang II or (and) PC/aPC was performed on HUVECs to detect changes in p66Shc, MMP2/9, and inflammatory markers (VCAM-1 and ICAM-1). PC/aPC significantly reduced the expression of p66Shc, MMP2/9, and inflammatory markers (VCAM-1 and ICAM-1) induced by Ang II. (D) Intervention with Ang II or (and) p66Shc-ShRNA was performed on HUVECs to detect changes in p66Shc, MMP2/9, and inflammatory markers (VCAM-1 and ICAM-1). p66Shc-ShRNA significantly reduced the expression of p66Shc, MMP2/9, and inflammatory markers (VCAM-1 and ICAM-1) induced by Ang II. (E) DHE staining was used to detect cell ROS production (red represents cell ROS production), and PC/aPC can significantly reduce Ang II induced ROS production. (F) Western blot detection of p66Shc protein expression in HUVECs after CHX and aPC intervention at different time points showed no significant change in p66Shc expression with or without aPC intervention. (G) RT-PCR detection of changes in p66Shc mRNA levels in HUVECs after AMD and aPC intervention at different time points showed no significant change in p66Shc mRNA levels with or without aPC intervention. (H) RT-PCR showed p66Shc mRNA expression levels after Ang II or (and) aPC intervention in HUVECs. The results showed that p66Shc mRNA levels were upregulated after Ang II intervention, and downregulated after aPC treatment. (I) Western blot shows the protein expression levels of p66Shc and AcH3 after Ang II or (and) aPC intervention in HUVECs. After Ang II intervention, the expression of p66Shc and AcH3 proteins was upregulated, and downregulated after aPC treatment on top of it. (All data represent the means ± SEM; *P < 0.05 vs. control, Ang II+PC group; ns indicates no statistically significant; One-way ANOVA).

To determine whether endothelial p66Shc actively contributes to AD pathology, we first confirmed that Ang II up-regulates p66Shc in HUVECs in a dose- and time-dependent manner (Figure 4A-B). Pre-treatment with PC or aPC markedly diminished Ang II-induced p66Shc, MMP-2/-9, ICAM-1, and VCAM-1 expression (Figure 4C; Supplementary Figure S1A). Silencing p66Shc with shRNA reproduced these anti-inflammatory and anti-remodelling effects, lowering MMP-2/-9 and adhesion-molecule levels (Figure 4D; Supplementary Figure S1B-D). Concordantly, dihydroethidium staining showed that PC/aPC curtailed the burst of ROS elicited by Ang II (Figure 4E).

Mechanistically, aPC reduced p66Shc transcript abundance rather than altering protein or mRNA stability, as indicated by cycloheximide and actinomycin-D chase assays (Figure 4F-G), whereas RT-qPCR demonstrated that aPC reversed Ang II-induced up-regulation of p66Shc mRNA (Figure 4H; Table S1). In our previous study aPC has been shown to modulate p66Shc mRNA expression epigenetically in diabetic nephropathy 12, thereafter we examined the key epigenetic event in the current study. In consistent with the previous study, Ang II increased histone-3 acetylation (AcH3), and this was normalized by aPC treatment (Figure 4I), indicating an epigenetic mechanism of p66Shc repression.

Since aPC exerts its downstream signaling transduction mainly *via* a PAR/EPCR dependent pathway, blocking experiments were applied to explore the receptor pathway. Indeed, an EPCR-neutralizing antibody abolished aPC-mediated reductions in both p66Shc and AcH3, whereas inhibiting PAR-1, PAR-2, PAR-3, or PAR-4 with blocking antibodies had no effect (Supplementary Figure S2A-B). At the same time, when we used PAR-related agonists, we found that the agonist of PAR1 could effectively reduce the expression of p66Shc induced by Ang II (Supplementary Figure S3A-B).

Taken together, these data show that aPC, acting through PAR1/EPCR-biased signalling, down-regulates p66Shc transcription epigentically, thereby suppressing downstream inflammatory, proteolytic, and oxidative responses in endothelial cells.

### 3.5 aPC prevents Ang II-driven mitochondrial trafficking of p66Shc, preserves membrane potential, and limits mitochondrial ROS

**Figure 5:**
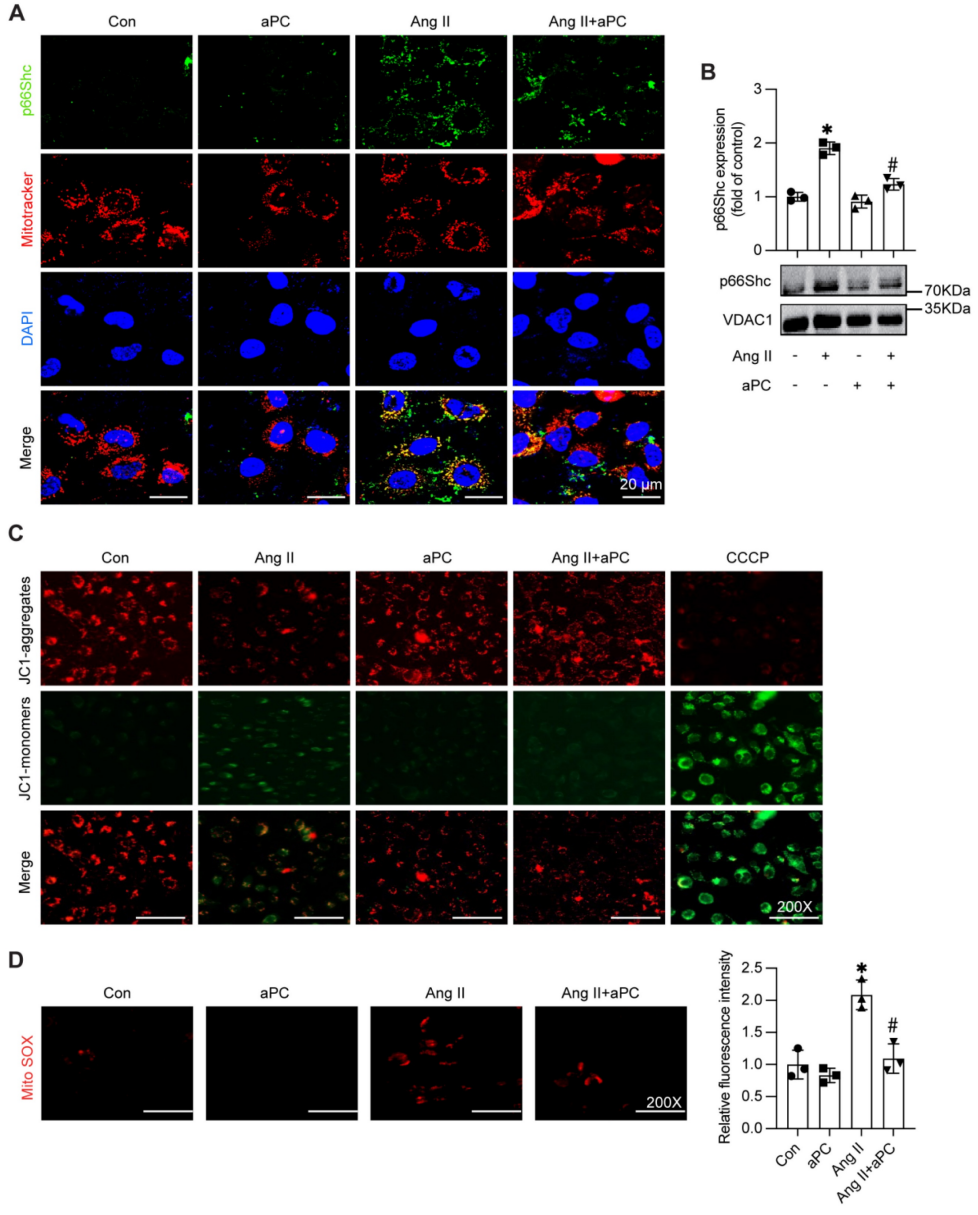
** aPC can reduce the mitochondrial translocation of p66Shc induced by Ang II, maintain the membrane potential of normal mitochondria, and thus reduce the production of mitochondrial ROS.** (A) Western blot detection of mitochondrial p66Shc protein translocation showed that Ang II can induce mitochondrial translocation of p66Shc, while aPC can inhibit its translocation. (B) p66Shc protein and MitoTracker cell immunofluorescence double staining (green represents p66Shc protein, red represents mitochondria, blue represents nucleus, and yellow represents p66Shc translocation into mitochondria) were used to detect mitochondrial translocation of p66Shc protein, and the results were consistent with Western blot results. Scale bar = 20 μm. (C) JC-1 staining (red represents the normal mitochondrial membrane potential, green represents the decline of mitochondrial membrane potential) detect the changes of mitochondrial membrane potential. Ang II can induce the decline of mitochondrial membrane potential, and aPC can inhibit the decline of its membrane potential. Scale bar: 200×. (D) Mito-Sox staining (red represents mitochondrial ROS) was used to detect the production of mitochondrial ROS in cells. The results showed that Ang II can promote mitochondrial ROS production, while aPC can inhibit its production. Scale bar: 200×. (All data represent the means ± SEM; *P < 0.05 vs. control, Ang II+PC group; two-way ANOVA).

Given that mitochondrial localization of p66Shc is a key trigger for oxidative injury, we examined whether aPC interferes with this trafficking step. Western blotting of isolated mitochondrial fractions showed a clear accumulation of p66Shc in Ang II-treated HUVECs, whereas aPC pre-treatment reduced mitochondrial p66Shc to near-baseline levels (Figure 5A; Supplementary Figure S4A). Whole-cell lysates confirmed that total p66Shc abundance was unchanged, indicating selective inhibition of mitochondrial import rather than altered protein synthesis (Figure 5B).

Functional assays corroborated these findings. JC-1 staining revealed that Ang II collapsed the mitochondrial membrane potential (ΔΨm), as evidenced by a shift from red J-aggregates to green monomers; aPC largely reduced the green/red ratio, indicating maintenance of ΔΨm (Figure 5C; Supplementary Figure S4B). Consistently, MitoSOX fluorescence demonstrated a substantial rise in mitochondrial superoxide after Ang II, which was markedly blunted by aPC (Figure 5D).

Collectively, these data show that aPC interrupts the Ang II-p66Shc axis at the level of mitochondrial translocation, thereby safeguarding mitochondrial function and suppressing ROS generation in endothelial.

### 3.6 aPC inhibits p66Shc mitochondrial import by promoting its O-GlcNAcylation at Thr29

**Figure 6:**
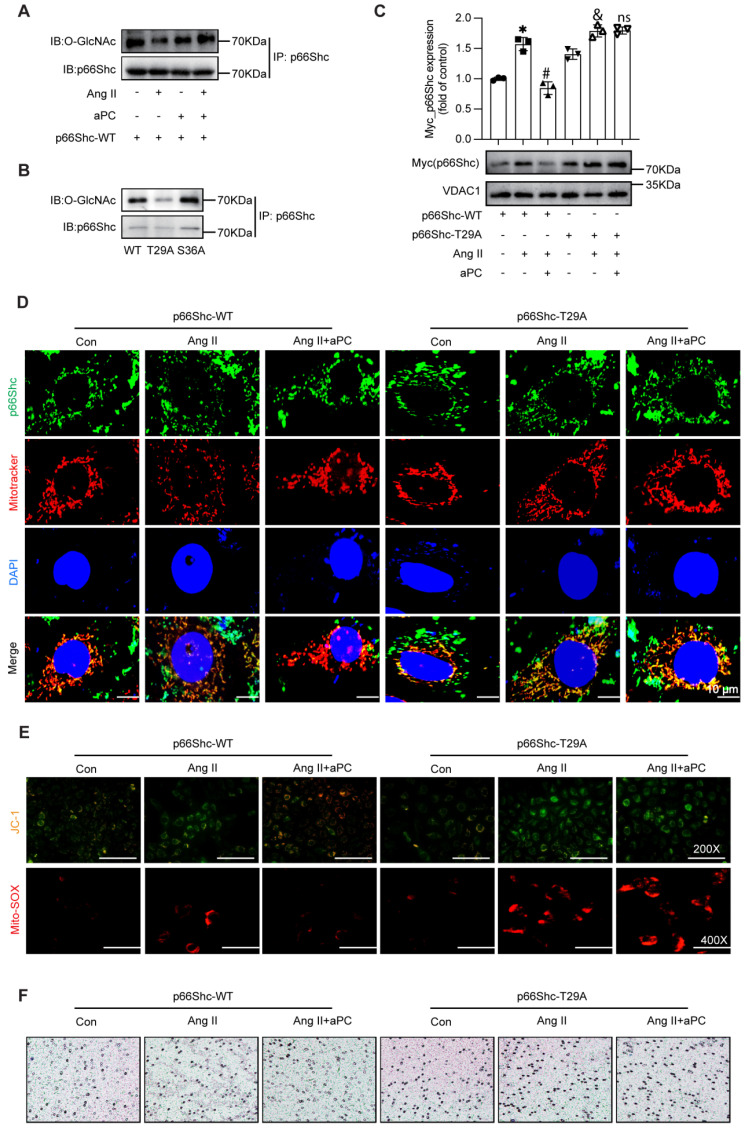
** aPC reduces mitochondrial translocation of p66Shc by increasing O-glycosylation modification of p66Shc.** (A) Immuno coprecipitation was used to detect the level of O-glycosylation of p66Shc under the intervention of Ang II or (and) aPC. Ang II can reduce the O-glycosylation of p66Shc, and aPC can increase the O-glycosylation. (B) 293T cells transfected with wild type high expression p66Shc plasmid and different mutant high expression p66Shc plasmid detected the level of O-glycosylation by immune coprecipitation. The level of O-glycosylation of the mutant plasmid at site 29 was lower than that of wild type, and the level of O-glycosylation of mutant plasmid at site 36 was not significantly changed compared with that of wild type. (C) Ang II or (and) aPC intervention in EA.hy 926 cells transfected with p66Shc-WT or p66Shc-T29A high expression plasmids detected mitochondrial translocation of p66Shc protein. aPC can inhibit Ang II induced mitochondrial translocation of p66Shc-WT, but cannot inhibit Ang II induced mitochondrial translocation of p66Shc-T29A. (D) By immunofluorescence double staining EA.hy926 cells transfected with p66Shc-WT or p66Shc-T29A high expression plasmids with p66Shc protein and MitoTracker (green represents p66Shc protein, red represents mitochondria, blue represents nucleus, and yellow represents p66Shc translocation into mitochondria), the mitochondrial translocation of p66Shc protein was detected. The results indicate that aPC can inhibit Ang II induced mitochondrial translocation of p66Shc-WT, but cannot inhibit Ang II induced mitochondrial translocation of p66Shc-T29A. Scale bar = 10 μm. (E) After transfection of p66Shc-WT or p66Shc-T29A high expression plasmids, EA.hy926 cells were stained with JC-1 (red represents normal mitochondrial membrane potential, green represents the decline of mitochondrial membrane potential) to detect the changes of mitochondrial membrane potential. aPC could inhibit the decrease of membrane potential caused by Ang II induced mitochondrial translocation of p66Shc-WT, but could not inhibit the decrease of mitochondrial membrane potential caused by Ang II induced mitochondrial translocation of p66Shc-T29A. Scale bar: 200×. After transfection of p66Shc-WT or p66Shc-T29A high expression plasmids, Mito-Sox staining (red represents mitochondrial ROS) was used to detect the production of mitochondrial ROS in cells. aPC could inhibit the decrease of mitochondrial ROS production caused by Ang II induced mitochondrial translocation of p66Shc-WT, but could not inhibit the decrease of mitochondrial ROS production caused by Ang II induced mitochondrial translocation of p66Shc-T29A. Scale bar: 400X. (F) Transwell experiment was used to detect the effect of EA. hy926 cells transfected with p66Shc-WT or p66Shc-T29A high expression plasmids on the migration of THP1 cells. Scale bar: 200X. (All data represent the means ± SEM; *P < 0.05 vs. p66Shc-WT group, Ang II+aPC+ p66Shc-WT group, Ang II+ p66Shc-T29A group; ns indicates no statistically significant; two-way ANOVA).

Because post-translational modifications of p66Shc govern its mitochondrial trafficking 25, 26, moreover O-GlcNAcylation contributes to mitochondrial dysfunction 27, we examined whether aPC alters p66Shc O-GlcNAcylation and subsequent mitochondrial trafficking. Immunoprecipitation with the pan-O-GlcNAc antibody (RL2) showed that Ang II markedly reduced O-GlcNAcylation of p66Shc, whereas aPC restored—and even surpassed—basal modification levels (Figure 6A). We detected the O-GlcNAcylation level of p66Shc in aortic specimens from both the control group and the AD group, and found that the O-GlcNAcylation level of p66Shc was decreased in the AD group (Supplementary Figure S11A).

In order to identify the critical O-GlcNAc site, we firstly tried the online prediction software. In-silico prediction (NetOGlyc 4.0) highlighted Thr29 and Ser36 as putative O-GlcNAc acceptors conserved across human, mouse and rat p66Shc (Supplementary Figure S5A). Secondly, mutagenesis assay was applied, mutating Thr29 or Ser36 to alanine in a p66Shc-overexpression construct revealed that only Thr29A (T29A) abolished aPC-induced O-GlcNAc, whereas Ser36A had no effect (Figure 6B; Supplementary Figure S5B). Subsequent functional consequences were examined in Ea.hy926 cells, Ang II triggered robust mitochondrial accumulation of wild-type p66Shc (WT), and aPC prevented this import. In contrast, aPC failed to block mitochondrial trafficking of the T29A mutant (Figure 6C). Immunofluorescence for p66Shc combined with MitoTracker confirmed these results (Figure 6D; Supplementary Figure S5C).

Consistently, aPC preserved mitochondrial membrane potential (JC-1) and suppressed mitochondrial superoxide (MitoSOX) in p66Shc-WT-transfected cells, but not in T29A-expressing cells (Figure 6E; Supplementary Figure S4D). Functionally, endothelial dysfunction reflected by THP-1 adhesion assays followed the same pattern: aPC reduced monocyte adhesion to WT-expressing endothelial cells, yet had no effect in T29A-expressing cells (Figure 6F; Supplementary Figure S5E).

Together, these data demonstrate that O-GlcNAcylation of p66Shc at Thr29 is both necessary and sufficient for aPC to block Ang II-induced mitochondrial import of p66Shc, thereby preserving mitochondrial function and limiting endothelial activation.

### 3.7. aPC promotes p66Shc O-glycosylation* via* the YB1/OGT axis

**Figure 7:**
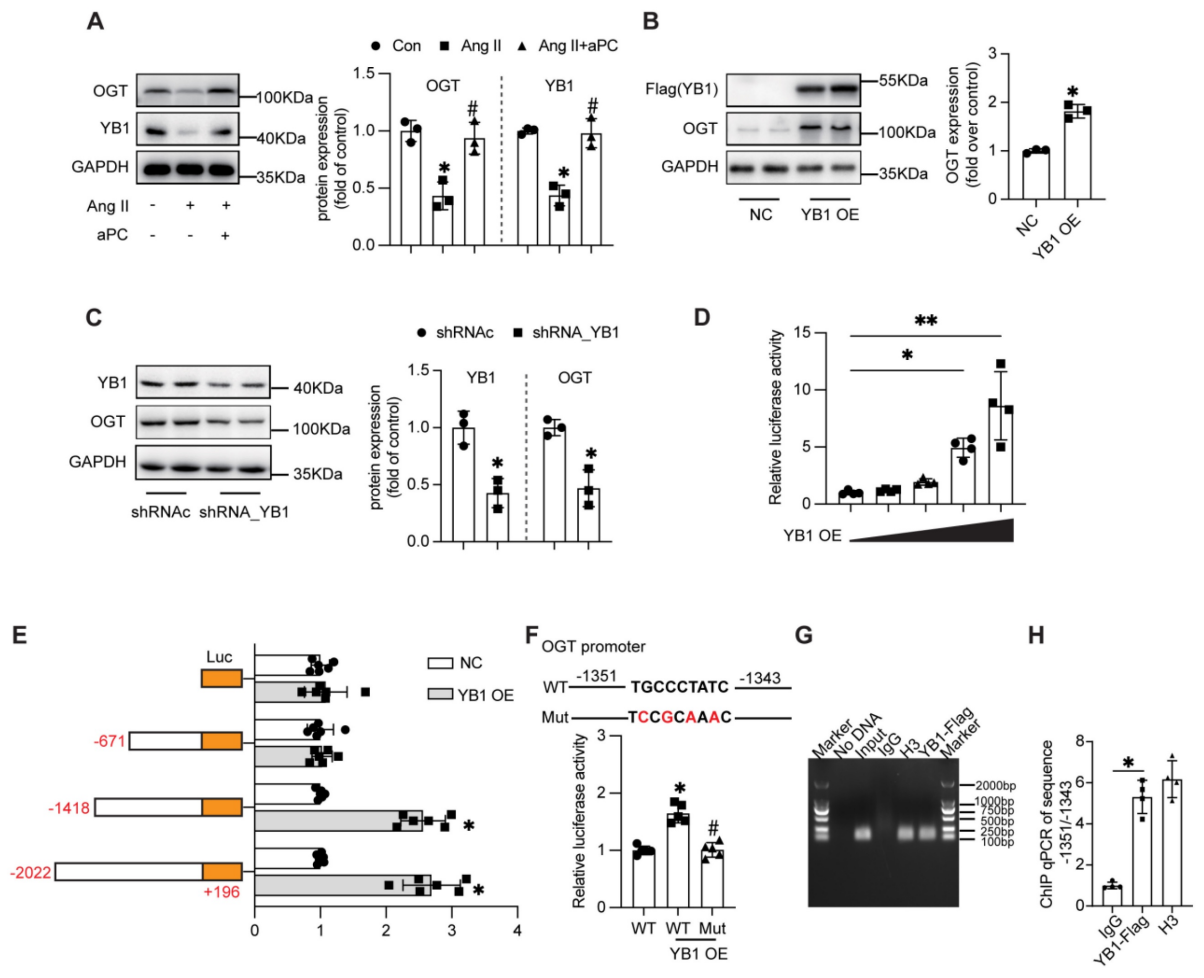
** aPC increases the O-glycosylation modification of p66Shc by regulating the YB1/OGT axis.** (A) Western blot detection of YB1 and OGT protein expression after Ang II or (and) aPC intervention in HUVECs cells showed that aPC could inhibit Ang II induced downregulation of OGT and YB1 protein expression. (B) Western blot detection of OGT expression in HUVECs cells transfected with highly expressed YB1 virus showed that after overexpression of YB1 protein, OGT protein was also highly expressed. (C) Western blot analysis showed that after transfection of YB1 shRNA into 293T cells, OGT protein expression was detected. The results showed that after inhibiting YB1 protein expression, OGT protein expression was also downregulated. (D) Luciferase activity of pGL3-OGT-promoter reporter plasmids stimulated by various YB-1 overexpression degree in 293T cells.(E)Luciferase activity of pGL3 (-2,022/+196), pGL3 (-1,418/+196), pGL3 (-671/+196), and pGL3-basic reporter plasmids stimulated by YB-1 overexpression in 293T cells. (F) Schematic illustration of mutant of the binding sites of YB-1 on OGT promoter in the predicted region of -1,351 to -1,343 (upper part). Luciferase activity was measured in 293T cells with mutant or wild type vector stimulated by YB-1 overexpression (lower part). (G-H) ChIP (chromatin immunoprecipitation) result was represented by agarose gel electrophoresis and real time qPCR which revealed the interaction of YB-1 and the promoter region of OGT.(All data represent the means ± SEM; *P < 0.05; Student's *t-test* or One-way ANOVA).

Because O-GlcNAcylation is catalysed by O-GlcNAc-transferase (OGT), we investigated whether aPC up-regulates OGT through the cold-shock transcription factor Y-box binding protein-1 (YB-1) 28. In HUVECs, Ang II markedly reduced YB-1 and OGT protein abundance, whereas aPC fully restored both proteins; EPCR blockade abolished this rescue (Figure 7A; Supplementary Figure S5). Mechanistically, aPC upregulates YB1 primarily via post-translational stabilization rather than transcriptional regulation or effects on mRNA stability (Supplementary Figure S8). We detected the expression levels of YB1 and OGT proteins in aortic specimens from both the control group and the AD group, and found that the expression levels of these two proteins were also decreased in the AD group (Supplementary Figure S11B).

Functional manipulation confirmed YB-1 as an upstream regulator of OGT: ectopic YB-1 increased, whereas YB-1 shRNA decreased, OGT protein levels (Figure 7B-C). Luciferase assays using serial deletions of the human OGT promoter revealed that YB-1 trans-activates the -2,022/+196 and -1,418/+196 fragments but not -671/+196, localizing the response element to -1,418/-671 bp (Figure 7D). In-silico motif prediction identified a TGCCCTATC sequence at -1,351/-1,343 bp; mutating this motif abolished YB-1-stimulated promoter activity, confirming it as the functional YB-1 binding site (Figure 7E). The regulatory effect of -1,351/-1,343 region was further determined by chromatin immunoprecipitation real time qPCR (Figure 7G-H).

Taken together, these data demonstrate that aPC, through PAR1/EPCR-biased signalling, sustains YB-1 expression, which in turn transcriptionally up-regulates OGT to elevate O-GlcNAcylation of p66Shc—the critical modification that blocks its mitochondrial import and curbs oxidative stress in aortic dissection.

## 4. Discussion

This study elucidates a novel protective mechanism by which aPC attenuates AD, highlighting the central role of epigenetic and post-translational regulation of p66Shc in mitochondrial oxidative stress. Clinically and experimentally, AD was characterized by reduced plasma aPC levels, increased expression of p66Shc, and elevated ROS production. *In vivo* experiments using both wild-type and TM^P/P^ mutant mice demonstrated that exogenous PC administration significantly reduced AD incidence, but only in mice with functional PC activation, underscoring the requirement for aPC in mediating vascular protection.

**Figure 8:**
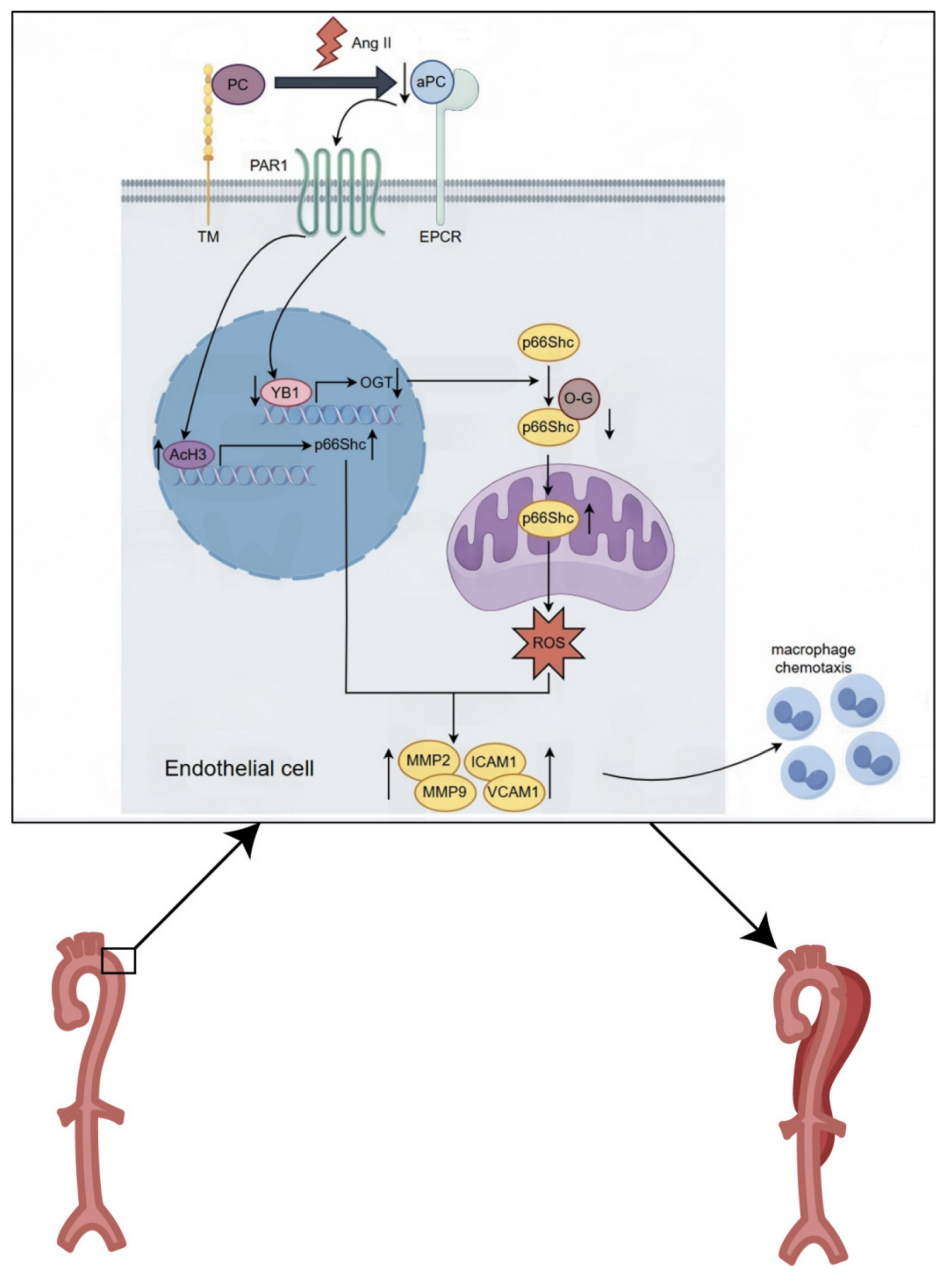
** Graphical abstract**. Mechanistically, we identified p66Shc as a downstream effector of aPC in both* in vivo* and *in vitro* models. Ang II-induced p66Shc upregulation was epigenetically suppressed by aPC *via* reduction of histone H3 acetylation at its promoter. Moreover, aPC inhibited the mitochondrial translocation of p66Shc by enhancing its O-glycosylation at threonine 29—a modification mediated *via* upregulation of the YB1/ OGT signaling axis. These events collectively mitigated mitochondrial ROS production and preserved mitochondrial membrane potential, contributing to the inhibition of AD onset and progression.

Mechanistically, we identified p66Shc as a downstream effector of aPC in both* in vivo* and *in vitro* models. Ang II-induced p66Shc upregulation was epigenetically suppressed by aPC *via* reduction of histone H3 acetylation at its promoter. Moreover, aPC inhibited the mitochondrial translocation of p66Shc by enhancing its O-glycosylation at threonine 29—a modification mediated *via* upregulation of the YB1/ OGT signaling axis. These events collectively mitigated mitochondrial ROS production and preserved mitochondrial membrane potential, contributing to the inhibition of AD onset and progression (Figure 8).

Mitochondrial dysfunction has been increasingly implicated in the pathogenesis of aortic aneurysms and dissections 29. Structural abnormalities in mitochondria, including swelling and vacuolization, have been observed in both human atherosclerotic aortae and elastase-induced aneurysmal models in mice 30, 31. Mitochondrial dysfunction has also been linked to age-associated arterial stiffening due to impaired mitophagy and biogenesis 32. Recent single-cell transcriptomic and metabolomic analyses of human and mouse aortic tissue further support the notion that mitochondrial impairment, particularly in VSMCs, is a hallmark of aneurysmal disease 33 34. Given the essential roles of mitochondria in ATP production, redox balance, and ROS regulation, mitochondrial integrity is critical for maintaining aortic wall homeostasis.

p66Shc has emerged as a pivotal regulator of mitochondrial ROS production and oxidative damage. Upon specific stimuli, p66Shc translocates to mitochondria, where it exacerbates ROS generation and promotes apoptosis 35. Sirt1-mediated deacetylation of lysine 81 and phosphorylation at serine 36 enhances p66Shc translocation and mitochondrial dysfunction in response to high glucose exposure 25. Similarly, propofol was shown to reduce LPS-induced oxidative damage in BEAS-2B cells by limiting PP2A-dependent p66Shc dephosphorylation and mitochondrial trafficking 36. Importantly, previous research has identified a cytoplasm-mitochondria signaling axis involving SIRT2, p66Shc, and mitochondrial ROS (mt ROS) as a critical regulator of vascular aging 37. Reduced SIRT2 activity with age leads to enhanced p66Shc activation, increased mROS production, and accelerated vascular senescence. Consistent with these findings, our study confirmed the mitochondrial localization of p66Shc under Ang II stimulation and demonstrated that aPC effectively inhibited this translocation, preserved mitochondrial membrane potential, and reduced ROS levels. Given this context, the elevated p66Shc activity observed in AD may reflect not only stress-induced mitochondrial dysfunction but also age-related dysregulation of the SIRT2-p66Shc pathway.

We further explored the regulatory mechanism by which aPC modulates p66Shc mitochondrial translocation. Prior studies have identified YB1 as a key transcriptional regulator downstream of aPC signaling *via* EPCR and PAR1 in diverse disease models 6. Our results indicate that aPC upregulates YB1 primarily via post-translational stabilization rather than transcriptional regulation or effects on mRNA stability. These findings are consistent with our previous work showing that aPC prevents YB1 degradation through a ubiquitin-proteasome-dependent mechanism in proximal tubular cells under hypoxia-reoxygenation 38 and in cardiomyocytes under hyperglycemic conditions 6. YB1 is a multifunctional cold shock protein involved in transcriptional and translational regulation, DNA repair, and chromatin remodeling 28. It can exert epigenetic control over downstream targets, including histone acetyltransferases and transcription factors. For example, YB1 promotes hyper-O-GlcNAcylation in hepatocellular carcinoma 39, enhances p300-mediated histone acetylation in 3D breast acini cultures 40, and facilitates ERα degradation *via* ubiquitination 41. Our data support the role of YB1 in transcriptional regulation of OGT under Ang II challenge, as YB1 overexpression increased OGT levels and luciferase reporter activity of the OGT promoter in a site-specific manner.

O-glycosylation is a dynamic and reversible post-translational modification critical for cellular responses to stress. It involves the addition of GlcNAc to serine/threonine residues *via* OGT. Under oxidative stress conditions, enhanced O-GlcNAcylation is protective, while its suppression exacerbates injury 42. In Parkinson's disease, aberrant glycosylation has been implicated in mitochondrial oxidative stress and inflammation 43. Similarly, CaMKII O-GlcNAcylation under hyperglycemic conditions promotes NOX2-mediated ROS generation 44, and downregulation of OGT alters mitochondrial architecture and function 45. In our study, mutation of the p66Shc O-glycosylation site (T29A) abolished the ability of aPC to prevent mitochondrial translocation, underscoring the functional significance of this modification in ROS regulation.

While comprehensive in scope and mechanistically detailed, our current study has several limitations. First, while our study identifies Thr29 O-GlcNAcylation of p66Shc as a critical regulatory mechanism, we did not perform *in vivo* rescue experiments or generate knock-in models to directly validate the functional relevance of this modification in aortic dissection. Future studies employing endothelial- or VSMC-targeted delivery of p66Shc mutants or Thr29 knock-in mouse models will be essential to confirm these mechanistic insights. Second, our work primarily focuses on endothelial cells, the role of p66Shc in vascular smooth muscle cells and potential intercellular interactions remains unaddressed. Third, although the human tissue data support our mechanistic findings, the sample size is limited and correlative in nature. Larger, independent patient cohorts will be required to validate the translational relevance of the aPC-YB1-OGT-p66Shc axis in aortic dissection. Fourth, our study focuses specifically on histone acetylation and O-glycosylation of p66Shc, but we did not further explore that reactive oxygen species can induce a wide spectrum of epigenetic changes beyond those examined here, including oxidative DNA lesions, alterations in histone methylation/acetylation, and modulation of chromatin accessibility 46. Last, several generalized therapeutic strategies—such as ACE inhibition, Ang II reduction, and antioxidant approaches—can improve mitochondrial function or reduce oxidative stress in vascular disease 47. However, these strategies act broadly on multiple pathways and may not specifically target the upstream molecular drivers that initiate mitochondrial dysfunction in AD.In contrast, the mechanism described in our study identifies a highly specific regulatory axis—aPC → YB1 → OGT → p66Shc O-GlcNAcylation—that directly modulates mitochondrial oxidative stress at its source.

Thrombotic complications in the setting of AD are indeed multifactorial. In AD, thrombosis within the false lumen is promoted by several mechanisms: (i) intimal tearing exposes subendothelial collagen and tissue factor, triggering platelet adhesion and aggregation; (ii) disturbed and slow blood flow with eddy currents in the false lumen favors thrombus formation; (iii) endothelial injury disrupts the balance between procoagulant and anticoagulant pathways; and (iv) inflammation activates coagulation while suppressing fibrinolysis, further enhancing thrombogenicity. In contrast, systemic PC/aPC administration is expected to counteract these processes by restoring anticoagulant signaling and exerting anti-inflammatory, endothelial-protective effects. In the present study, although we did not perform a systematic quantitative analysis of thrombus burden within the false lumen, our mechanistic framework and the cited literature strongly support the notion that TM deficiency favors, whereas PC/aPC supplementation mitigates, thrombotic complications in the context of AD.

Collectively, our findings reveal that aPC attenuates mitochondrial oxidative stress in AD by dual mechanisms: (1) transcriptional repression of p66Shc *via* epigenetic histone deacetylation and (2) inhibition of p66Shc mitochondrial translocation through YB1/OGT-mediated O-glycosylation. These mechanisms converge to suppress ROS accumulation and protect vascular integrity.

## Supplementary Material

Supplementary methods, figures and tables.
