# Supplementary material for: Epigenetic and O-glycosylation regulation of p66Shc mitigates mitochondrial oxidative stress in aortic dissection
Source: Theranostics. 2026 Feb 4;16(8):4394–410. doi: 10.7150/thno.124508 (PMC12906141; doi:10.7150/thno.124508)
Supplement: Supplementary file 1 — Supplementary methods, figures and tables. [file thnov16p4394s1.pdf]

Supplementary Materials

Materials

The following antibodies were used in the current study: Rabbit polyclonal antibodies to AcH3, ICAM-1, VCAM-1,MMP2/9,SHC1,OGT, were from ABclonal Technology; rabbit polyclonal antibody to YB-1 and Acetyl-lys was from CUSABIO; rabbit monoclonal antibody to YB-1 was from Abcam;, mouse monoclonal antibodies to IgG, flag, Myc, O-GlcNAc, rabbit polyclonal antibody to ubiquitin,SUMO1,SUMO2/3 were from Cell Signaling Technology; rabbit polyclonal antibodies to PAR1, PAR2, PAR3, PAR4, EPCR,SHC1were from Santa Cruz; rabbit polyclonal antibody to GAPDH, rabbit anti-mouse IgG-HRP conjugated secondary antibody and mouse anti-rabbit IgG-HRP conjugated secondary antibody were from Servicebio.

The following reagents were used in the current study: streptozotocin, MG132, D-(+)-Glucose solution, D-Mannitol were from Sigma-Aldrich; fetal bovine serum, low glucose DMEM, DMEM were from Gibco; protein A/G agarose beads was from Santa Cruz; protease inhibitor cocktail and phosphatase inhibitor cocktail were from MedChemExpress; RNAiso Plus was from Takara; antibiotic solution, enhanced chemiluminescence reagent, BCA reagent were from BORTER Biological Technology; PVDF membrane was from Millipore GmbH; transfection reagent Turbofect was from Thermo Fisher Scientific; p66Shc ORF overexpression plasmid, shRNA vectors for YB-1 and SHC1 were from Thermo scientific and OriGene, respectively; Restriction Enzymes, T4 DNA ligase, cycloheximide were from New England Biolabs; DAB substrate Kit for peroxidase was from Vector Laboratories; ABScript II reverse transcription premix and Genious 2X SYBR Green Fast qPCR Mix were from ABclonal Technology; lepirudin was from celgene; Dual luciferase assay was from Promega; ChIP assay kit, mouse or human activated protein C ELISA Kits were from CUSABIO.

Figure S1

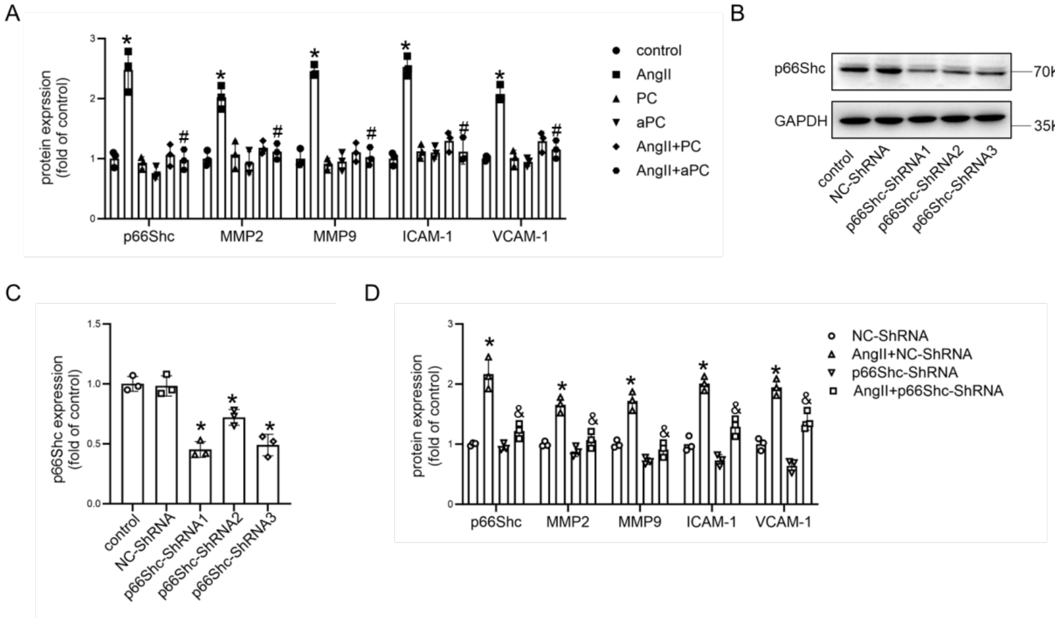

Figure S1: (A) Scatter plot results of p66Shc, MMP2/9, and inflammatory markers (VACM-1 and ICAM-1) in HUVECs intervented with Ang II or (and) PC/aPC. (B) The expression of p66Shc decreases in HUVECS infected with p66Shc-ShRNA lentivirus. (C) Scatter plot results of p66Shc in HUVECs infected with p66Shc-ShRNA lentivirus. (D) Scatter plot results of p66Shc, MMP2/9, and inflammatory markers (VACM-1 and ICAM-1) in HUVECs intervented with Ang II or (and) p66Shc-ShRNA lentivirus. Data were represented as fold of control, mean ± SD. $^{*}P<0.05$ vs control, $^{\#}P<0.05$ vs Ang II+aPC, $^{\&}P<0.05$ vs Ang II+p66Shc-ShRNA. Student's *t* test or one-way ANOVA, Bonferroni comparison test.

Figure S2

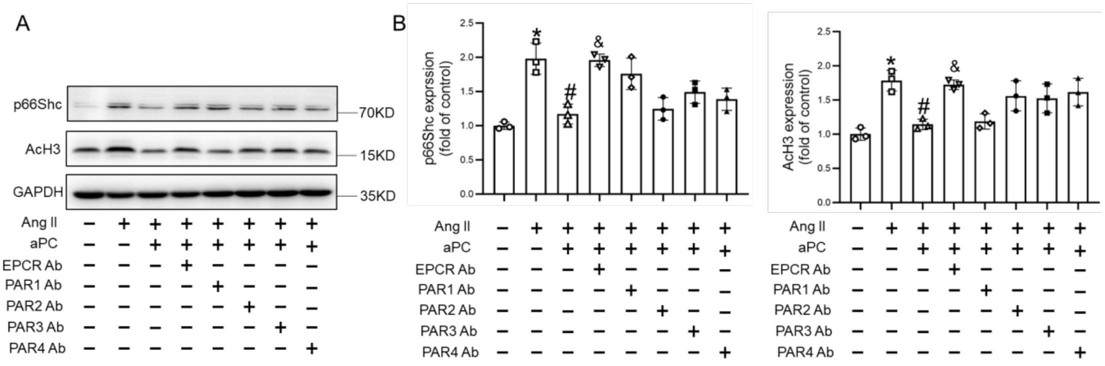

Figure S2: aPC significantly reduced p66Shc and AcH3 expression via EPCR. (A) HUVECs were pre-treated with specific blocking antibodies (20 μg/ml, 1 h) then aPC (20 nM, 30 min), and thereafter Ang II (10^{-5} M, 24 h). Western blots showed that EPCR blocking antibodies abolished the protective effect of aPC on reducing the expression of p66Shc and AcH3. (B) Scatter plot results of p66Shc and AcH3 in HUVECs pre-treated with specific blocking antibodies (20 μg/ml, 1 h) then aPC (20 nM, 30 min), and thereafter Ang II (10^{-5} M, 24 h). Data were represented as fold of control, mean ± SD. $^{*}P<0.05$ vs control, $^{\#}P<0.05$ vs Ang II, $^{\&}P<0.05$ vs Ang II+aPC. Student's *t* test or one-way ANOVA, Bonferroni comparison test.

Figure S3

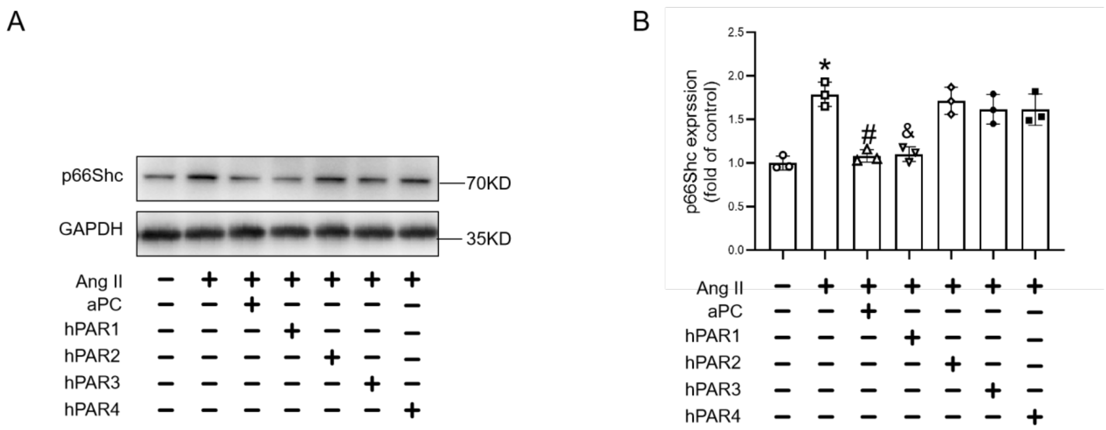

Figure S3: PAR1 agonist significantly reduced p66Shc induce by Ang II. (A) HUVECs were pre-treated with PAR agonist (1 mg/ml, 1 h) or aPC (20 nM, 30 min), and thereafter Ang II (10^{-5} M, 24 h). Western blots showed that PAR1 agonist significantly reduced p66Shc induce by Ang II.. (B) Scatter plot results of p66Shc in HUVECs pre-treated with PAR agonist (1 mg/ml, 1 h) or aPC (20 nM, 30 min), and thereafter Ang II (10^{-5} M, 24 h). Data were represented as fold of control, mean ± SD. **P*<0.05 vs control, $^{\#}P<0.05$ vs Ang II, $^{\&}P<0.05$ vs Ang II+aPC. Student's *t* test or one-way ANOVA, Bonferroni comparison test.

Figure S4

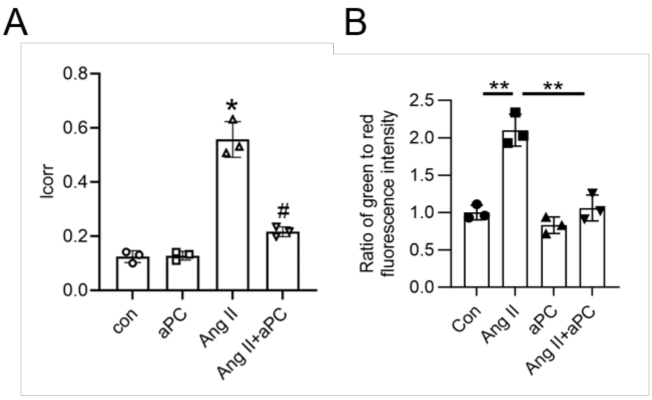

Figure S4: (A) Scatter plot analysis of Icorr (colocalization correlation index) values between p66Shc and mitochondria in HUVECs treated with Ang II and/or aPC, based on immunofluorescence double staining using p66Shc antibody and MitoTracker. (B) Scatter plot analysis of JC-1 staining results in HUVECs treated with Ang II and/or aPC, reflecting changes in mitochondrial membrane potential..

Figure S5

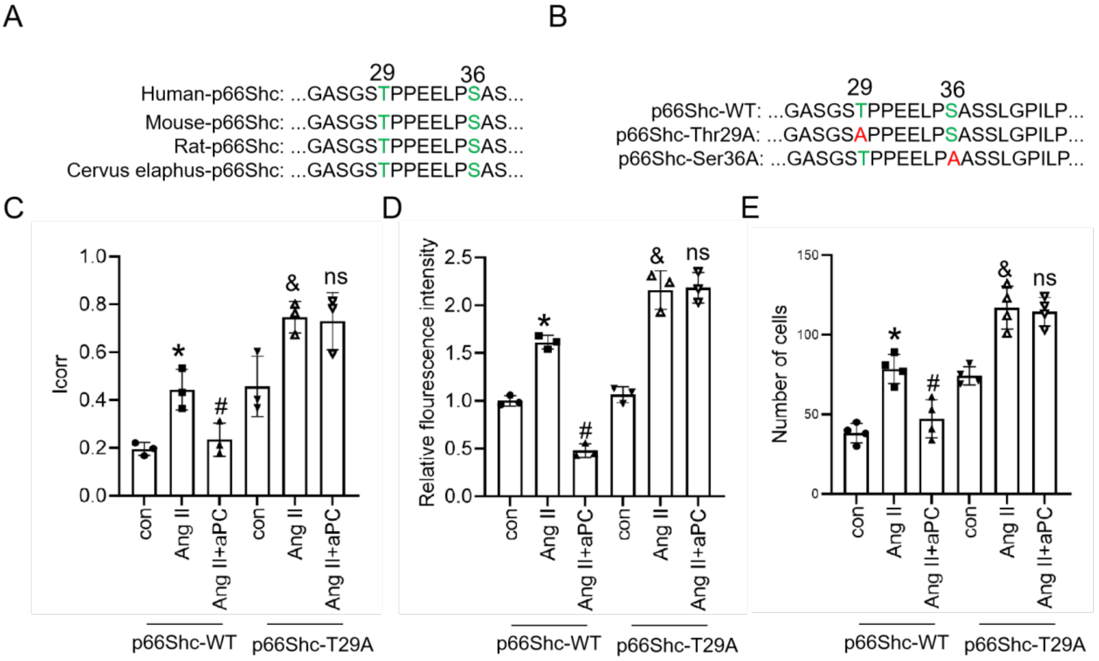

Figure S5: (A) Human, mouse, and rat derived p66Shc proteins all share a common site 29 threonine and site 36 serine that may undergo O-glycosylation modification. (B) Plasmids with high expression of p66Shc protein underwent mutations at different sites, with threonine at site 29 and serine at site 36 mutated into alanine. (C) Scatter plot results of immunofluorescence double staining EA.hy926 cells transfected with p66Shc-WT or p66Shc-T29A high expression plasmids with p66Shc protein and MitoTracker. (D) Scatter plot results of Mito-Sox staining. (E) Scatter plot results of transwell experiment that was used to detect the effect of EA. hy926 cells transfected with p66Shc-WT or p66Shc-T29A high expression plasmids on the migration of THP1 cells. Data were represented as fold of control, mean ± SD. *P<0.05 vs con+p66Shc-WT, $^{\#}P$<0.05 vs Ang II+p66Shc-WT, $^{\&}P$<0.05 vs Ang II+p66Shc-T29A ns indicates no statistically significant. Student's *t* test or one-way ANOVA, Bonferroni comparison test.

Figure S6

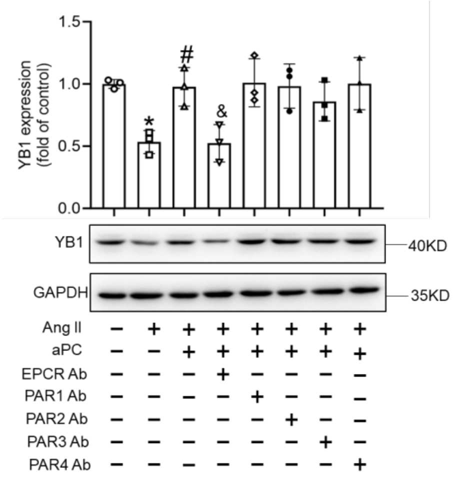

Figure S6: HUVECs were pre-treated with specific blocking antibodies (20 μg/ml, 1 h) then aPC (20 nM, 30 min), and thereafter Ang II (10^{-5} M, 24 h). Western blots showed that EPCR blocking antibodies abolished the protective effect of aPC on maintaining the expression of YB1. (B) Scatter plot results of YB1 in HUVECs pre-treated with specific blocking antibodies (20 μg/ml, 1 h) then aPC (20 nM, 30 min), and thereafter Ang II (10^{-5} M, 24 h). Data were represented as fold of control, mean ± SD. **P*<0.05 vs control, $^{\#}$*P*<0.05 vs Ang II, $^{\&}$*P*<0.05 vs Ang II+aPC. Student's *t* test or one-way ANOVA, Bonferroni comparison test.

Figure S7

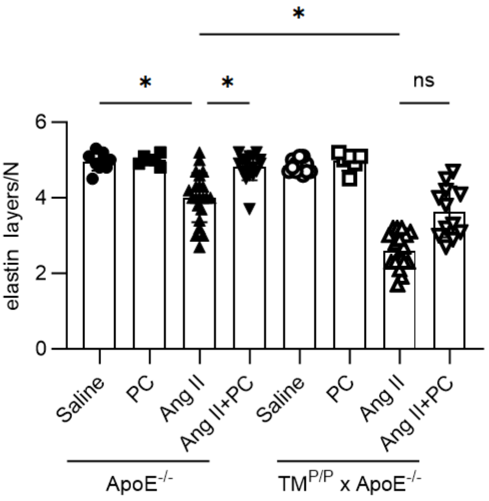

Figure S7.quantitative analysis of elastic fiber integrity**.** All data represent the means ± SEM, n≥6; *P<0.05 vs. saline group, Ang II+PC group in wild-type mice, Ang II+PC group in TMP/P type mice; ns indicates no statistically significant; One-way ANOVA)

Figure S8

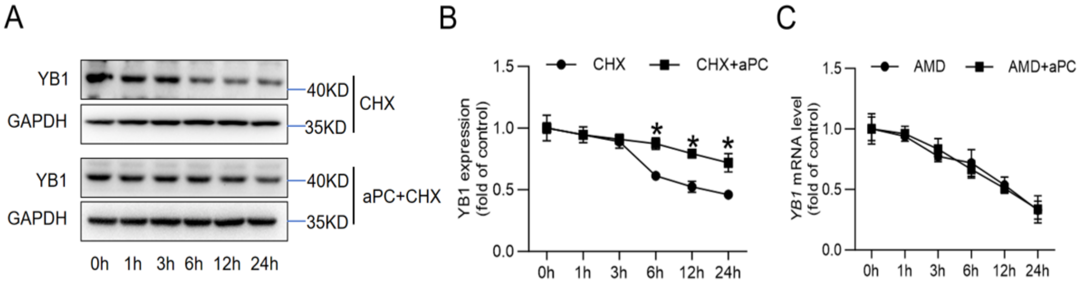

Figure S8**. aPC stabilizes YB1 protein by inhibiting its degradation.** (A–B) Western blot analysis of YB1 protein levels in HUVECs treated with cycloheximide (CHX) in the presence or absence of aPC at indicated time points. aPC markedly attenuated CHX-induced YB1 protein degradation, indicating increased protein stability. (C) Quantitative RT-PCR analysis of YB1 mRNA levels in HUVECs treated with actinomycin D (AMD) with or without aPC showed no significant differences over time, indicating that aPC does not alter YB1 mRNA stability. All experiments were performed in triplicate.

Figure S9

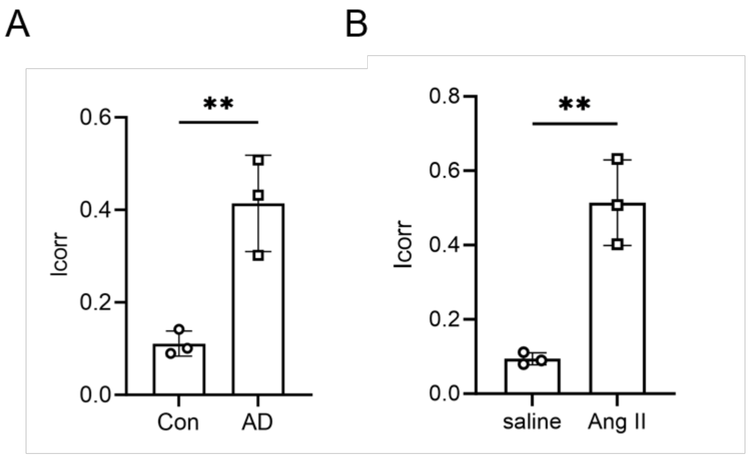

Fgiure S9.(A-B)Scatter plot results of immunofluorescence double staining human and mouse aortae with the p66Shc protein and the endothelial cell marker CD31 protein.

Figure S10

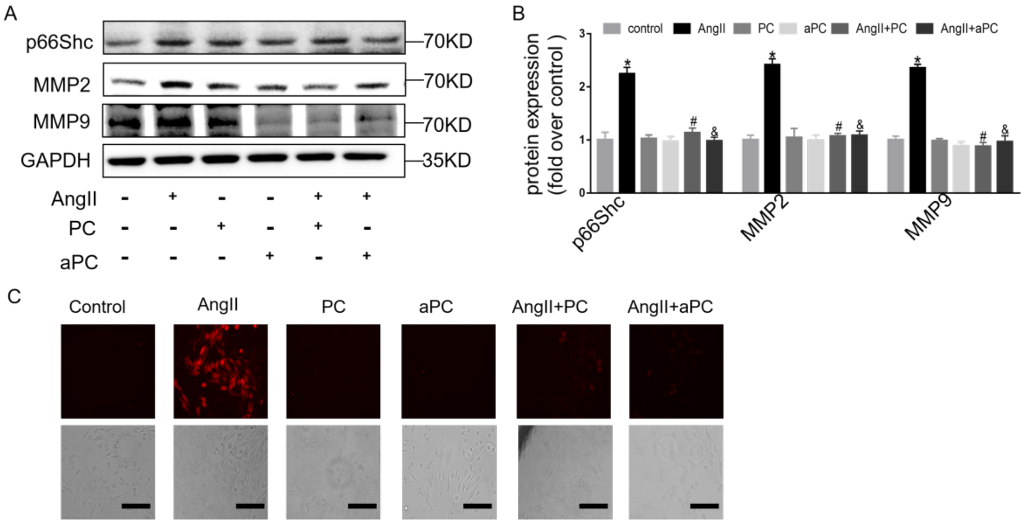

Figure S10. aPC inhibits Ang II–induced p66Shc expression, MMP2/9 upregulation, and oxidative stress in VSMCs. (A–B) Western blot analysis of VSMCs treated with Ang II and/or PC/aPC showing changes in p66Shc and MMP2/9 expression. PC/aPC markedly attenuated the Ang II–induced increase in p66Shc and MMP2/9 levels.(C) DHE staining was used to assess intracellular ROS production (red fluorescence). PC/aPC significantly reduced Ang II–induced ROS generation in VSMCs. All data represent mean ± SEM. *P < 0.05 vs. control or Ang II+PC group; ns, not statistically significant; one-way ANOVA.

Figure S11

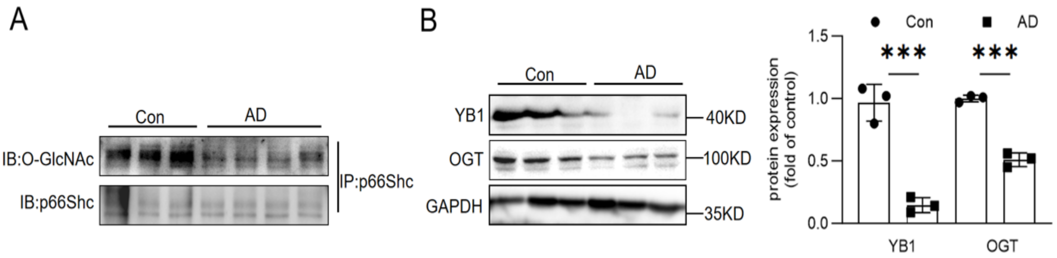

Figure S11. O-glycosylation of p66Shc and expression of YB1 and OGT are reduced in aortic tissue from patients with aortic dissection. (A) Immunoprecipitation analysis of human aortic samples showing decreased O-glycosylation of p66Shc in patients with aortic dissection compared with controls. (B) Western blot analysis demonstrating reduced YB1 and OGT protein expression in aortic dissection samples. All data represent mean ± SEM. ****P* < 0.001; Student's *t*-test.

Table S1:Baseline Data

	Control group （n=27）	AD group (n=29)	*p*-value
Sex			*p＞0.05*
Male	17	20	
Female	10	9	
Age,(Mean,years)	54.96	55.48	*p*＞0.05
Hypertension,(%)	77.78%	82.76%	*p*＞0.05
Diabetes, (%)	18.52%	20.69%	*p*＞0.05
Smoking, (%)	22.22%	24.14%	*p*＞0.05
Drinking alcohol, (%)	29.63%	27.59%	*p*＞0.05
ACEI/ARB Drugs	59.26%	%65.52	*p*＞0.05

Table S2: Primer sequences used for real time quantitive PCR to detect relative mRNA levels in HUVECs.

Gene	Forward	Reverse
SHC1	GCCAAAGACCCTGTGAATCAG	GTATTGTTTGAAGCGCAACTCG
GAPDH	CCCTTAAGAGGGATGCTGCC	TACGGCCAAATCCGTTCACA
YBX1	TAGACGCTATCCACGTCGTAG	ATCCCTCGTTCTTTTCCCCAC

Table S3: Primer sequences used for ChIP.

Gene	Forward	Reverse

OGT promoter	CCAGCATGTCCCTGAGTTATTAAAC	CCAGTTGAATGCAGACACCTT
